# ZUP1 contributes to cisplatin resistance and treg-related signaling in non–small cell lung cancer

**DOI:** 10.1038/s41598-026-70626-y

**Published:** 2026-09-10

**Authors:** Wanyan Xiong, Zhixuan Deng, Fang Ding, Zhike Xiao, Wang Shi, Gang Gu, Nan Yan

**Affiliations:** 1 Department of Pulmonary and Critical Care Medicine, The Affiliated Nanhua Hospital, University of South China, Hengyang, Hunan China; 2 Institute of Cell Biology, Hengyang Medical School, University of South China, Hengyang, 421000 Hunan China; 3 The Second Affiliated Hospital, University of South China, Hengyang, Hunan China

**Keywords:** Non–small cell lung cancer (NSCLC), Cisplatin resistance, ZUP1 (ZUFSP), Regulatory T cells (Tregs), Peimisine, Cancer, Immunology, Oncology

## Abstract

Acquired cisplatin resistance limits platinum chemotherapy efficacy in non–small cell lung cancer (NSCLC) and is accompanied by tumor immune microenvironment (TME) remodeling. We integrated public NSCLC single-cell RNA-seq datasets to compare cellular composition and CellChat-inferred communication between post-chemotherapy remission and relapse/resistant tumors, and validated ZUP1 expression and prognostic relevance in TCGA and external cohorts. In cisplatin-resistant A549/R and SK-MES-1/R cells, we generated stable ZUP1 knockdown and assessed proliferation, tumorsphere growth and formation, apoptosis (Annexin V/PI) and BAX/BCL2 expression, and xenograft growth. Tumor cell–peripheral Treg co-cultures were used to quantify the proportion of FOXP3⁺ cells within the gated CD4⁺ population. Natural-product screening was guided by molecular docking, and ZUP1–compound binding was evaluated by surface plasmon resonance (SPR). Peimisine responses were compared between parental and cisplatin-resistant cells. In A549/R cells, experimentally measured viability across a peimisine–cisplatin concentration matrix and cisplatin dose–response curves at fixed peimisine concentrations were used to evaluate changes in cisplatin sensitivity. Resistant tumors exhibited remodeled cellular composition and enhanced intercellular communication, including increased epithelial-to-Treg signaling. ZUP1 was upregulated in resistant models and associated with unfavorable prognosis. ZUP1 knockdown suppressed growth and tumorsphere formation, increased apoptosis, reduced tumorigenicity in vivo, and reduced the proportion of CD4⁺FOXP3⁺ Treg-like cells in co-culture. Peimisine directly bound ZUP1 by SPR, inhibited resistant cell growth, and was associated with lower estimated cisplatin IC₅₀ values in A549/R cells at fixed peimisine concentrations. ZUP1 contributes to the maintenance of cisplatin-resistant growth and is associated with enhanced Treg-related immunosuppressive signaling in NSCLC. Peimisine directly binds ZUP1 and may serve as a preliminary natural-product lead for further target-validation and combination studies.

**Supplementary Information:**

The online version contains supplementary material available at https://doi.org/10.1038/s41598-026-70626-y.

## Introduction

Non–small cell lung cancer (NSCLC) accounts for approximately 80%–85% of all lung cancers and remains one of the leading causes of cancer-related mortality worldwide^1^. Although targeted therapies and immunotherapies have improved outcomes in selected patient populations, platinum-based chemotherapy continues to be a key component of the NSCLC treatment paradigm and is widely used across multiple clinical settings^2^. However, once resistance to platinum chemotherapy develops, tumors often exhibit persistent progression, diminishing benefit from subsequent treatments, and limited survival gains, thereby representing a major barrier to durable therapeutic efficacy^3^. Therefore, elucidating the critical molecular nodes that drive chemoresistance and developing actionable strategies to overcome it remain of clear scientific and clinical importance.

Growing evidence indicates that the origin of chemoresistance is not determined solely by tumor cell–intrinsic alterations, but is closely linked to dynamic remodeling of the tumor immune microenvironment (TME/TIME)^4,5^. Under therapeutic pressure, interaction networks between tumor cells and immune cells are reprogrammed, fostering immunosuppressive niches that blunt drug efficacy and stabilize resistant phenotypes.^6,7^ Platinum agents act not only on tumor cells but also influence non-malignant components and immune states, thereby reshaping cellular composition and functional programs within the TME^8^. In this context, “microenvironment remodeling driven by tumor–immune crosstalk” has become an important entry point for understanding and therapeutically targeting chemotherapy resistance.

The magnitude, directionality, and persistence of information exchange between tumor and immune cells are, to a large extent, finely tuned by post-translational modifications (PTMs). Prior studies have systematically shown that PTMs regulate the stability, localization, and functional outputs of immune-related molecules—including immune checkpoints and their upstream/downstream signaling components—thereby affecting immune recognition, immune evasion, and therapeutic responses^9^. Among diverse PTMs, reversible regulatory systems composed of E3 ligases and “erasers” (e.g., deubiquitinases, DUBs) are characterized by strong signal amplification, well-defined nodes, and druggability^10–12^. Dysregulation of these key enzymes may alter tumor-cell signaling presentation/secretion and receptor–ligand interactions, shifting tumor–immune communication in a coordinated manner, promoting immunosuppressive microenvironmental shaping, and contributing to resistance development^13,14^.

From a translational perspective, directly targeting PTM-controlling enzymes within tumor cells to reprogram the immune microenvironment represents an actionable anti-resistance strategy. Traditional medicine and natural products provide an important source of lead compounds for this approach. Recent reviews have highlighted that traditional Chinese medicine–derived agents and natural products can modulate multi-target networks, influence inflammation- and hypoxia-related microenvironments, and intervene in resistance-associated pathways, thereby showing potential to overcome platinum resistance within a modern framework centered on “target–pathway–evidence” chains^15,16^. Accordingly, screening natural monomers capable of targeting key PTM enzymes and applying them to improve TME states and enhance chemosensitivity constitutes a research path with both biological rationale and translational feasibility.

In this study, we integrated single-cell and public-cohort analyses to identify the PTM-related enzyme ZUP1 as a candidate node associated with chemoresistance in NSCLC, and linked it to enhanced immune-related signaling output from epithelial cells and increased Treg-associated communication (e.g., SPP1 and MHC-II pathways). In cisplatin-resistant cell models, ZUP1 knockdown inhibited proliferation and tumorsphere formation, promoted apoptosis, and reduced tumor burden in xenograft assays. Moreover, the natural compound peimisine bound ZUP1, suppressed the growth of resistant cells, and was associated with lower estimated cisplatin IC₅₀ values in A549/R cells under the tested combination conditions.Collectively, these findings support a translational concept: targeting PTM key enzymes in tumor cells to remodel the immune microenvironment as a strategy to mitigate chemotherapy resistance.

## Materials and methods

### Single-cell RNA-seq analysis

Public NSCLC single-cell transcriptomic datasets were used to investigate the association between ZUP1 and the tumor immune microenvironment. Single-cell RNA sequencing (scRNA-seq) data were obtained from the GEO database (GSE207422 and GSE243013), representing post-chemotherapy remission and relapse/resistant NSCLC tumor samples, respectively^17,18^. After downloading the raw expression matrices, integrated analyses were performed using R (v4.2.2) and Seurat (v4.3.0). Quality control criteria were as follows: cells with < 200 or > 5,000 detected genes and cells with > 10% mitochondrial gene expression were excluded. After filtering, data were normalized using the LogNormalize method (scale factor = 10,000), and the top 2,000 highly variable genes were selected for downstream analyses. Principal component analysis (PCA) was performed on the normalized expression matrix, and significant principal components were selected based on the elbow plot to construct the nearest-neighbor graph and perform clustering (Seurat FindClusters, resolution = 0.5). Uniform Manifold Approximation and Projection (UMAP) was used for two-dimensional visualization.

Cell types were annotated according to the expression patterns of canonical marker genes (e.g., EPCAM for epithelial cells, CD3D for T cells, CD68 for macrophages, and FOXP3 for Tregs), with additional reference to the TISCH database (v1.0; accessed August 2025) for tumor single-cell annotations and marker gene information to facilitate manual validation and consistency checks. Differential expression analysis was conducted within major cell populations to compare transcriptional differences between resistant and remission groups. In addition, tumor epithelial cells were stratified by ZUP1 expression (ZUP1-high vs. ZUP1-low), and differentially expressed genes were identified using the Wilcoxon rank-sum test. Gene Ontology (GO) and Kyoto Encyclopedia of Genes and Genomes (KEGG) enrichment analyses were performed using clusterProfiler (v4.6.2).

Cell–cell communication analysis was performed using CellChat (v1.5.0), which infers communication networks in the tumor microenvironment based on ligand–receptor interactions. CellChat objects were constructed separately for resistant and remission conditions using the default human ligand–receptor database, and differences in the total number of interactions, interaction strength, and pathway-level information flow were compared. Outgoing signaling from ZUP1-high tumor epithelial cells to Tregs was quantified, and interaction axes that were more active in the resistant context (e.g., SPP1–CD44) were identified. Communication results were visualized using bubble plots and circos networks.

### Public transcriptomic and prognostic data analysis

To evaluate the prognostic relevance of ZUP1 in NSCLC, gene-expression and clinical follow-up data were collected from six publicly available NSCLC cohorts derived from TCGA, OncoSG, CPTAC, and CAS, comprising a total of 1,283 patients. Expression data were normalized and log2-transformed when necessary. After harmonization of the overall survival data, the cohorts were combined for pooled survival analysis. Overall survival was evaluated using Kaplan–Meier curves and the log-rank test. Hazard ratios (HRs) and 95% confidence intervals (CIs) were estimated using Cox proportional hazards regression in R.

### Cell culture and reagents

Human non–small cell lung cancer (NSCLC) cell lines A-549 (lung adenocarcinoma; RRID: CVCL_0023) and SK-MES-1 (lung squamous cell carcinoma; RRID: CVCL_0630) were purchased from the American Type Culture Collection (ATCC; Manassas, VA, USA). All human cell lines used in this study are reported using the official Cellosaurus name and Research Resource Identifier (RRID) as available in the ExPASy Cellosaurus database. Cisplatin-resistant sublines (A549/R and SK-MES-1/R) were established according to previously published methods by continuous selection under stepwise increasing concentrations of cisplatin for approximately 6 months. All cell lines were authenticated by short tandem repeat (STR) (or SNP) profiling within the last three years and confirmed to be free of mycoplasma contamination. A549 cells were cultured in Kaighn’s F-12 K medium (Gibco, Thermo Fisher Scientific) supplemented with 10% fetal bovine serum (FBS). SK-MES-1 cells were cultured in minimum essential medium (MEM; Gibco, Thermo Fisher Scientific) supplemented with 10% FBS. All media contained penicillin (100 U/mL) and streptomycin (100 µg/mL). Cells were maintained at 37 °C in a humidified incubator with 5% CO₂ and passaged at 70–80% confluence. Peimisine (HPLC purity > 98%) was purchased from Sigma-Aldrich (St. Louis, MO, USA). Cisplatin was also obtained from Sigma-Aldrich and prepared as a 1 mg/mL stock solution in normal saline. Unless otherwise specified, all other chemical reagents were of analytical grade and purchased from Sigma-Aldrich or Thermo Fisher Scientific.

### RNA interference (shRNA knockdown of ZUP1)

To stably silence human ZUP1 (Gene ID: 221302), multiple ZUP1-targeting shRNAs were cloned into a U6-driven GFP-lentiviral vector, with a non-targeting shRNA as control (sh-Scr) (Table S1). Lentiviruses were produced in HEK293T cells using a standard three-plasmid system, and viral supernatants were collected, centrifuged, and 0.45 μm-filtered. A549/R and SK-MES-1/R cells were transduced (with polybrene when needed), and medium was replaced after 12–16 h. At ~ 72 h post-infection, GFP-positive cells were enriched by FACS to generate stable shZUP1 and sh-Scr populations. Knockdown efficiency of the candidate shRNAs was initially screened at the mRNA level by qRT–PCR. The selected shZUP1 construct was subsequently validated at the protein level by Western blotting before use in downstream functional assays.

### Quantitative real-time PCR (qRT–PCR)

Total RNA was extracted using TRIzol Reagent (Invitrogen, Thermo Fisher Scientific) and quantified (A260/280) with a NanoDrop or equivalent. Equal amounts of RNA were reverse-transcribed into cDNA and analyzed by qRT–PCR using a SYBR Green system with a two-step cycling program. Each sample was run in technical replicates with at least three independent biological replicates. No-template controls (NTC) and, when required, no–reverse transcription (–RT) controls were included. Melt-curve analysis was used to confirm amplification specificity. Primer sequences are provided in Supplementary Table 1 (Table S1).Gene expression was normalized to GAPDH and calculated using the 2^−ΔΔCt method. ZUP1 knockdown efficiency was normalized to sh-Scr, and other targets were normalized to the corresponding control group. Data are presented as mean ± SD.

### Western blotting

Cells and xenograft tumor tissues were lysed in RIPA buffer containing protease inhibitors, and protein concentrations were determined using a BCA assay. Equal amounts of protein were separated by SDS–PAGE and transferred onto PVDF membranes. After blocking with 5% non-fat milk for 1 h, the membranes were incubated overnight at 4 °C with antibodies against ZUP1/ZUFSP (Invitrogen, Cat. No. PA5-24149; 1:1,000), BAX (Proteintech, Cat. No. 50599-2-Ig; 1:5,000), BCL2 (Proteintech, Cat. No. 12789-1-AP; 1:2,000), GAPDH (Proteintech, Cat. No. 60004-1-Ig; 1:10,000), or β-Tubulin (Proteintech, Cat. No. 66240-1-Ig; 1:20,000). The membranes were then incubated with HRP-conjugated secondary antibodies (Proteintech, Cat. Nos. SA00001-1 or SA00001-2; 1:5,000) for 1 h at room temperature. Protein bands were visualized using enhanced chemiluminescence and quantified using ImageJ. Protein expression was normalized to GAPDH or β-Tubulin. Three independent experiments were performed.

### Candidate factor expression and SPP1 ELISA

A549/R and SK-MES-1/R cells stably expressing sh-Scr or sh-ZUP1 were cultured under identical experimental conditions. Total RNA was extracted, and the mRNA expression levels of SPP1, CXCL16, and PTGS2 were measured by qRT–PCR. GAPDH was used as the internal reference gene, and relative gene expression was calculated using the 2^−ΔΔCt method.

For measurement of secreted SPP1, conditioned medium was collected from the indicated cell cultures and clarified by centrifugation to remove cells and cellular debris. SPP1 concentrations were measured using a Human Osteopontin/OPN Quantikine ELISA Kit (R&D Systems, Bio-Techne, Minneapolis, MN, USA; Cat. No. DOST00) according to the manufacturer’s instructions. Viable cell numbers were determined at the time of supernatant collection, and the measured SPP1 concentrations were normalized to 1 × 10^6^ viable cells. Three independent biological experiments were performed.

### Tumorsphere formation assay

To assess 3D growth and tumorsphere-forming capacity, stable cells (sh-Scr and shZUP1) were dissociated, counted, and seeded at 1 × 10^3 cells/well in ultra-low attachment 6-well plates (Corning). Cells were cultured in serum-free tumorsphere medium consisting of DMEM/F12 (Gibco) supplemented with 1× B27 (Thermo Fisher Scientific), EGF (20 ng/mL), bFGF (20 ng/mL), and 1% methylcellulose to minimize cell aggregation. Cultures were maintained at 37 °C with 5% CO₂ for ~ 10 days. Spheres with a diameter ≥ 50 μm were counted under an inverted microscope. The diameter (d) of each tumorsphere was measured from microscopic images using ImageJ, and tumorsphere volume was estimated assuming spherical geometry using the formula V = πd³/6. Sphere-forming efficiency was calculated as (number of spheres/number of seeded cells) ×100%. At least three technical replicates were included per group, and experiments were repeated independently at least three times.

### Apoptosis assay by annexin V/PI flow cytometry

Apoptosis was assessed using Annexin V/PI double staining followed by flow cytometry. Stable cells (sh-Scr and shZUP1) were treated as indicated, harvested by trypsinization, and combined with culture supernatants to maximize recovery of apoptotic cells. Cells were washed twice with cold PBS, resuspended in 1× binding buffer (BD Biosciences), and stained with FITC–Annexin V and PI (BD Pharmingen) for 15 min at room temperature in the dark. After adding binding buffer, samples were immediately analyzed on a BD FACSCanto II flow cytometer. Data were processed using FlowJo (v10.8); debris and doublets were excluded, and cells were classified as viable (Annexin V^−/PI^−), early apoptotic (Annexin V^+/PI^−), or late apoptotic/necrotic (Annexin V^+/PI^+). At least 10,000 events were acquired per sample. Experiments included ≥ 3 biological replicates and were repeated independently at least three times.

### Tumor cell–treg co-culture and flow-cytometric analysis

PBMCs were obtained from healthy donors with ethical approval and informed consent. CD4^+CD25^+ Tregs were enriched by magnetic sorting (Miltenyi Biotec) and confirmed by flow cytometry (CD4^+FOXP3^+ >90%). Stable A549/R or SK-MES-1/R cells (sh-Scr or shZUP1) were seeded in 6-well plates overnight and co-cultured with Tregs at the indicated ratios in RPMI-1640 containing 10% FBS and IL-2 (100 IU/mL) for 48 h. Suspended cells were collected and stained for CD4/CD25 and intracellular FOXP3 (eBioscience Foxp3/Transcription Factor Staining Buffer Set). Data were acquired on a BD FACSCanto II and analyzed with FlowJo to quantify CD4^+FOXP3^+ Tregs and compare activation-related readouts between groups.

### Molecular docking and virtual screening

A human ZUP1 structure was obtained from the PDB and prepared for docking. A public natural-product library was standardized and docked to the predicted ZUP1 binding pocket using AutoDock Vina. Compounds were ranked by predicted binding affinity, and top hits were visually inspected. Peimisine was selected for subsequent SPR validation and functional assays.

### Surface plasmon resonance (SPR)

Direct binding between peimisine and ZUP1 was assessed by SPR on a Biacore T200 (Cytiva). Recombinant human ZUP1 was immobilized on a CM5 chip by amine coupling, with a reference channel for background subtraction. Peimisine was injected as a concentration series in HBS-EP + at 25 °C, and sensorgrams were fitted using a 1:1 Langmuir model to estimate the dissociation constant (K_d). Experiments were repeated at least three times.

### Drug treatment and cisplatin dose–response analysis

Parental A549 and SK-MES-1 cells and their corresponding cisplatin-resistant derivatives, A549/R and SK-MES-1/R, were seeded in 96-well plates and treated with the indicated concentrations of peimisine. Cell viability was measured using the CCK-8 assay and normalized to the corresponding vehicle-treated control.For the combination experiment, A549/R cells were treated with a two-dimensional concentration matrix of peimisine and cisplatin for the indicated duration. Cell viability for each experimentally tested concentration combination was normalized to the untreated control and is presented as the mean of three independent biological experiments.To evaluate cisplatin responses at fixed peimisine concentrations of 0, 30, and 50 µM, viability under combination treatment was normalized within each independent experiment to the corresponding peimisine-only condition according to the following equation: conditional viability (%) = viability of the peimisine–cisplatin combination/viability of the matched peimisine-only treatment ×100. Cisplatin dose–response curves were fitted using a constrained four-parameter log-logistic model, with the lower and upper asymptotes constrained to 0% and 100%, respectively.

### Xenograft tumor model

All animal procedures were approved by the Animal Ethics Committee of the University of South China. Female BALB/c nude mice (BALB/c-nu/nu, 4–5 weeks old) were housed under SPF conditions. To assess the in vivo effects of ZUP1 knockdown, A549/R cells stably expressing sh-Scr or shZUP1 were injected subcutaneously into the right flank/axillary region (5 × 10^6 cells per mouse; *n* = 3 per group) in PBS mixed 1:1 with Matrigel (Corning; total volume 100 µL). Tumor length and width were measured every 3–4 days, and volume was calculated as V = (length × width^2)/2. Tumor growth and body weight were monitored, and mice were euthanized at predefined humane endpoints. Tumors were excised and weighed; portions were fixed in 10% neutral buffered formalin for histology/IHC, and the remainder was snap-frozen in liquid nitrogen for molecular analyses. Measurements and endpoint assessments were performed in a blinded manner when feasible.

### Statistical analysis

Quantitative data are presented as mean ± SD unless otherwise indicated. Statistical analyses were performed using GraphPad Prism and/or R. Comparisons between two groups were conducted using two-tailed Student’s t-tests, and multiple-group comparisons were analyzed by one-way ANOVA with appropriate post hoc multiple-comparison corrections when needed. Time-course proliferation and tumor growth curves were analyzed by two-way ANOVA. Survival was evaluated using Kaplan–Meier analysis with the log-rank test. A two-sided *P* < 0.05 was considered statistically significant. All experiments were performed with at least three independent biological replicates, and details are provided in the figure legends.

## Results

### Integrated single-cell transcriptomic analysis reveals differences in cellular states and predicted communication between resistant and remission NSCLC

**Fig. 1:**
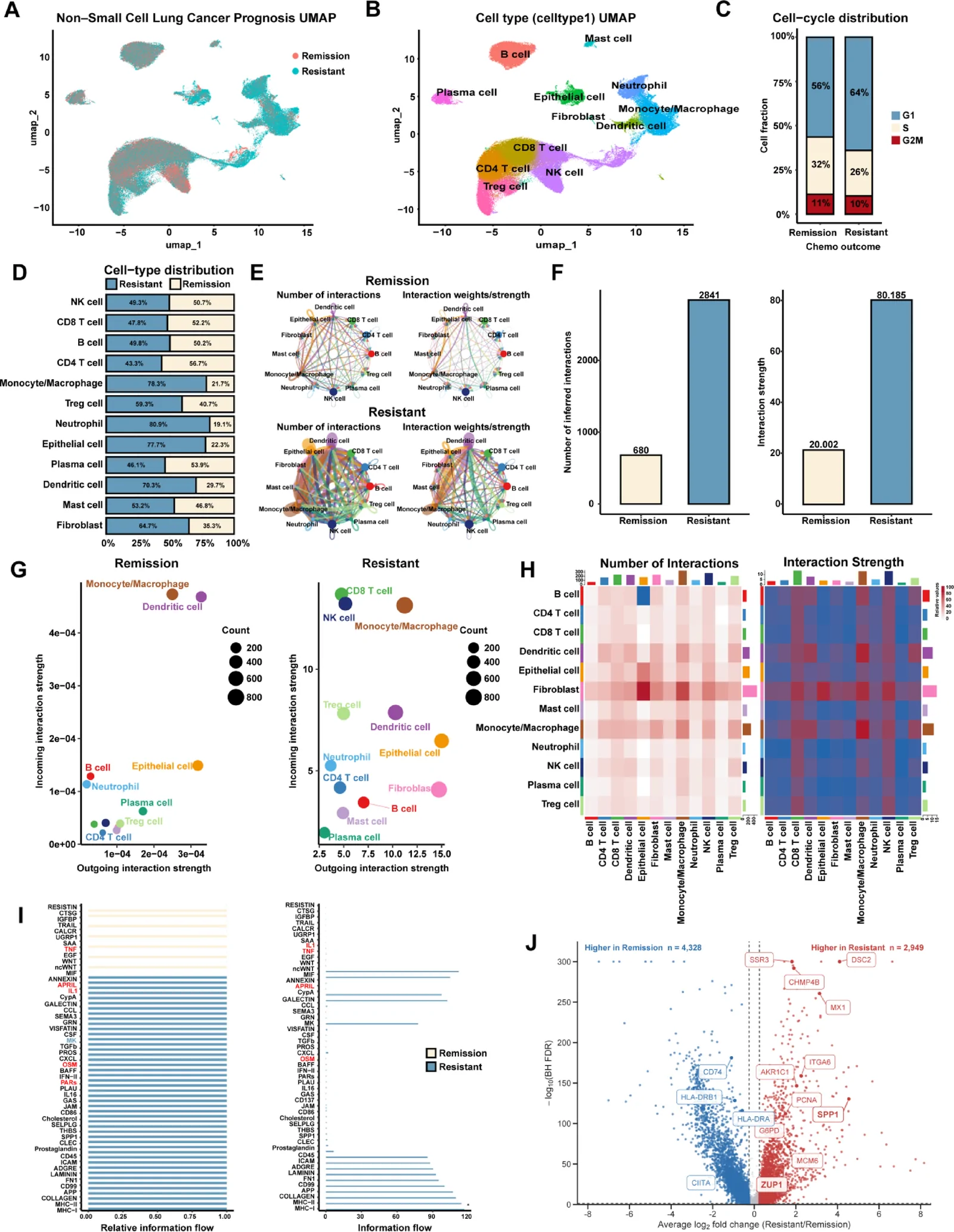
Integrated single-cell transcriptomic profiling reveals differences in cellular composition and intercellular communication between post-treatment resistant and remission NSCLC. (**A**) Uniform manifold approximation and projection (UMAP) of all cells retained after quality control and data integration, colored according to clinical outcome as Remission or Resistant. (**B**) UMAP visualization of the integrated dataset colored by the annotated major cell populations, including B cells, plasma cells, CD4⁺ T cells, CD8⁺ T cells, regulatory T cells, NK cells, monocytes/macrophages, dendritic cells, neutrophils, mast cells, epithelial cells, and fibroblasts. Cell identities were assigned according to canonical lineage markers and reference annotations. (**C**) Cell-cycle phase distribution in the Remission and Resistant groups. Stacked bars show the proportions of cells assigned to the G1, S, and G2M phases within each clinical-outcome group. (**D**) Distribution of cells from the Resistant and Remission groups within each annotated cell population. Percentages indicate the relative contribution of the two clinical-outcome groups to each cell population based on the pooled single-cell dataset. (**E**) CellChat-inferred intercellular communication networks in the Remission and Resistant groups. Circle plots show the predicted number of ligand–receptor interactions and the aggregated interaction strength among the major cell populations. Edge width represents the inferred number or strength of interactions between the corresponding sender and receiver populations. (**F**) Comparison of the total number of inferred interactions and the total aggregated interaction strength between the Remission and Resistant groups. (**G**) Scatter plots showing the outgoing and incoming communication strengths of each major cell population in the Remission and Resistant groups. The x-axis represents outgoing interaction strength, the y-axis represents incoming interaction strength, and node size represents the inferred number of interactions involving the corresponding cell population. (**H**) Heatmaps showing the inferred number of interactions and aggregated interaction strength for individual sender–receiver cell-population pairs. Rows represent signaling sender populations, columns represent receiver populations, and color intensity indicates the magnitude of the predicted communication. (**I**) Comparison of pathway-level information flow between the Remission and Resistant groups. The left panel shows the relative contribution of each group to the corresponding signaling pathway, whereas the right panel shows the absolute inferred information flow. (**J**) Volcano plot showing differentially expressed genes in epithelial cells between the Resistant and Remission groups. Positive log₂ fold-change values indicate higher expression in Resistant epithelial cells, whereas negative values indicate higher expression in Remission epithelial cells. Red and blue points denote genes significantly enriched in the Resistant and Remission groups, respectively; gray points indicate genes that did not meet the prespecified differential-expression thresholds. Differential expression was assessed using the Wilcoxon rank-sum test with multiple-testing correction.

To characterize differences between post-treatment Resistant and Remission NSCLC, we integrated single-cell transcriptomic datasets representing these two clinical outcomes. UMAP visualization showed distinct distributions of cells from the Resistant and Remission groups within the integrated embedding, indicating differences in their cellular composition and transcriptional states (Fig. 1A–B). Cell-cycle scoring showed that the Resistant group contained a higher relative proportion of G1-phase cells and lower proportions of S- and G2M-phase cells than the Remission group (Fig. 1C). Because this analysis was performed across all included cells, it does not establish an epithelial-cell-specific proliferative phenotype. Analysis of cellular composition further showed that the relative contributions of the Resistant and Remission groups varied across the annotated cell populations (Fig. 1D). However, the relative distributions of CD8⁺ T cells and NK cells were broadly comparable between the two groups, and no clear reduction in these populations was evident. Collectively, these findings indicate differences in overall cell-cycle distribution and cellular composition between Resistant and Remission NSCLC.

We next inferred intercellular communication patterns using CellChat. Compared with the remission group, resistant tumors showed a marked increase in both the total number of interactions and overall interaction strength, forming a denser and more highly connected communication network with richer cross-lineage connectivity (Fig. 1E–F). Incoming and outgoing signaling analyses further indicated that epithelial cells in resistant tumors shifted from being relatively “responsive” to becoming prominent communication hubs: they exhibited stronger outbound signaling while simultaneously receiving more feedback signals from myeloid and stromal compartments (Fig. 1G). At the level of specific cell–cell pairs, the connectivity and weights of multiple interaction axes—including epithelial↔myeloid/dendritic, epithelial↔fibroblast, and myeloid↔T-cell interactions—were elevated in resistant tumors, suggesting an overall intensification of coupling between tumor cells and the TME (Fig. 1H). At the pathway level, information flow was globally increased in the resistant group, with enhanced signaling concentrated in adhesion/ECM remodeling, chemotaxis and immune regulation, antigen presentation, and lipid metabolism pathways (Fig. 1I).

Finally, differential-expression analysis identified transcriptional differences between epithelial cells from the Resistant and Remission groups (Fig. 1J). Genes showing higher expression in Resistant epithelial cells included those annotated to processes related to cell proliferation, epithelial and extracellular-matrix organization, vesicular transport and secretion, and metabolic regulation, whereas a subset of genes with lower expression was associated with immune-related processes. The complete list of differentially expressed genes, together with the average log₂ fold change, nominal P value, adjusted P value, direction of change, and functional annotation, is provided in Supplementary Tables S2–S4. Collectively, these analyses reveal differences in cellular composition and predicted intercellular communication between post-treatment Resistant and Remission NSCLC. The stronger predicted signaling output from epithelial cells provided a basis for subsequent evaluation of candidate factors associated with epithelial–immune communication.

### Multimodal analyses identify ZUP1 as a candidate DUB associated with NSCLC resistance

**Fig. 2:**
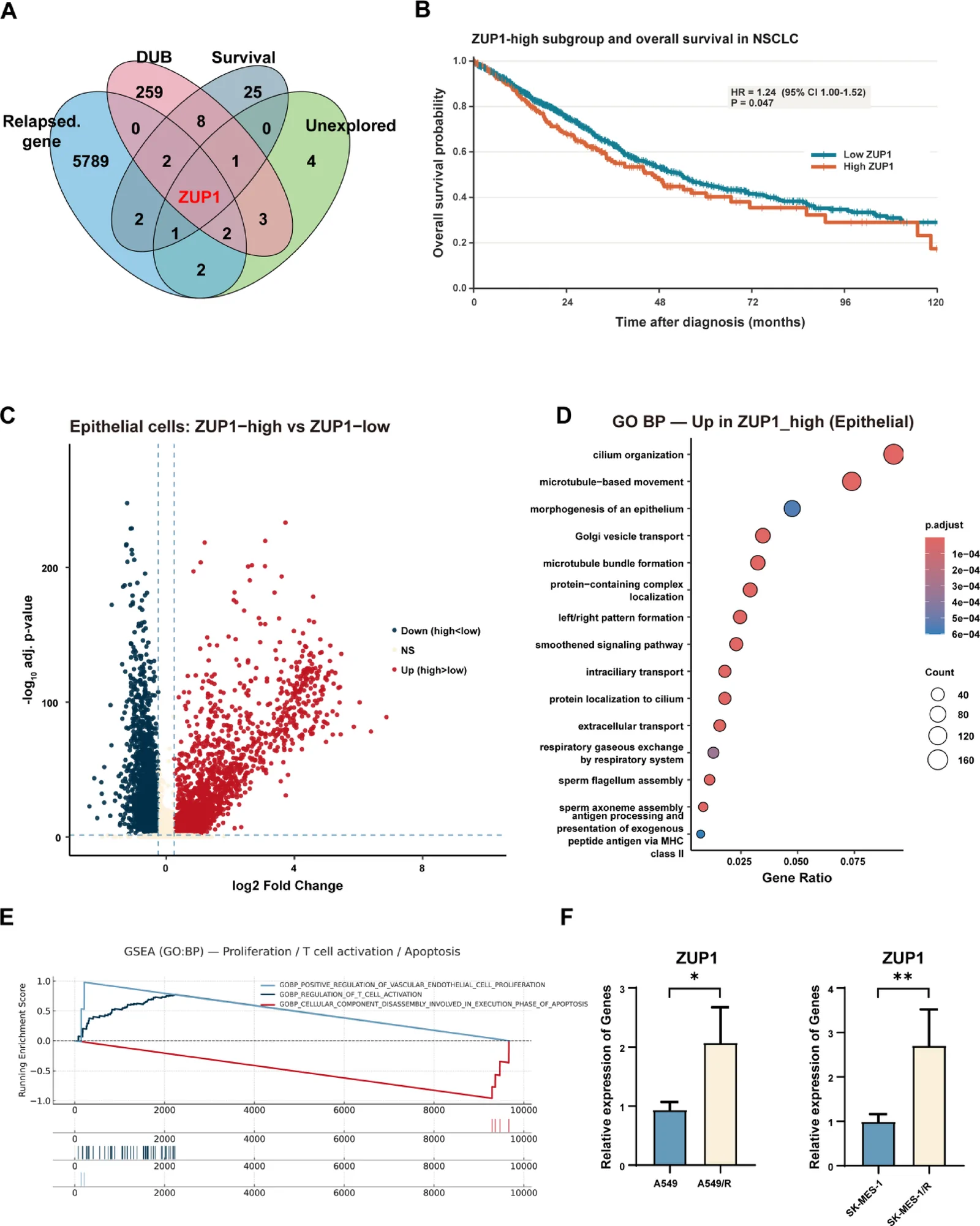
Multimodal screening identifies ZUP1 as a candidate deubiquitinase associated with cisplatin-resistant NSCLC. (**A**) Venn diagram integrating relapse/resistance-associated genes, a curated deubiquitinase gene set, survival-associated genes, and genes classified as previously unexplored according to the screening criteria described in the Methods. ZUP1 was identified as the common candidate at the intersection of the four gene sets. (**B**) Kaplan–Meier analysis of overall survival according to ZUP1 expression in a pooled NSCLC cohort comprising six publicly available datasets from TCGA, OncoSG, CPTAC, and CAS, with a total of 1,283 patients. Patients were stratified into ZUP1-high and ZUP1-low groups using the median ZUP1 expression level as the cutoff. High ZUP1 expression was associated with poorer overall survival than low ZUP1 expression (HR = 1.24, 95% CI = 1.00–1.52; log-rank P = 0.047). Tick marks indicate censored observations. (**C**) Volcano plot showing differentially expressed genes between ZUP1-high and ZUP1-low epithelial cells in the integrated single-cell dataset. Positive log₂ fold-change values indicate higher expression in ZUP1-high epithelial cells, whereas negative values indicate higher expression in ZUP1-low epithelial cells. Red and dark-blue points represent significantly upregulated and downregulated genes, respectively; nonsignificant genes are shown in gray. Dashed lines indicate the prespecified fold-change and adjusted-P-value thresholds. (**D**) Gene Ontology Biological Process enrichment analysis of genes significantly upregulated in ZUP1-high epithelial cells. Dot size represents the number of genes assigned to each term, the x-axis indicates the gene ratio, and dot color represents the adjusted P value. (**E**) Gene set enrichment analysis showing representative gene sets associated with cell proliferation, regulation of T-cell activation, and the execution phase of apoptosis in ZUP1-high versus ZUP1-low epithelial cells. Running enrichment scores and the positions of genes belonging to each gene set within the ranked gene list are shown. (F) Quantitative real-time PCR analysis of ZUP1 mRNA expression in parental A549 and SK-MES-1 cells and their corresponding cisplatin-resistant derivatives, A549/R and SK-MES-1/R. Expression was normalized to GAPDH and calculated using the 2−∆∆Ctmethod, with the corresponding parental cell line used as the reference.Data in (**F**) are presented as mean ± SD from three independent biological experiments. Statistical significance was assessed using a two-tailed unpaired Student’s t-test. ∗P < 0.05;∗∗P < 0.01.

To identify clinically relevant candidate targets at the level of “key enzymes regulating post-translational modifications” that may contribute to NSCLC resistance, we established a multimodal intersection-based screening framework integrating relapse-associated gene sets, a curated DUB gene list, prognosis-associated genes, and genes that have not been systematically investigated. This integrative strategy prioritized ZUP1 as the top candidate within the intersecting set (Fig. 2A). To evaluate the prognostic relevance of ZUP1, we performed a pooled survival analysis using six publicly available NSCLC cohorts from TCGA, OncoSG, CPTAC, and CAS, comprising a total of 1,283 patients. Kaplan–Meier analysis showed that patients with high ZUP1 expression had poorer overall survival than those with low ZUP1 expression (HR = 1.24, 95% CI = 1.00–1.52; log-rank *P* = 0.047; Fig. 2B).

At single-cell resolution, we further focused on tumor epithelial cells and stratified them by ZUP1 expression. Compared with ZUP1-low cells, ZUP1-high epithelial cells exhibited extensive transcriptional reprogramming, with differential expression analysis showing broad upregulation of genes under the ZUP1-high condition (Fig. 2C). Functional enrichment of upregulated genes demonstrated significant enrichment in ciliogenesis/microtubule-based movement, epithelial morphogenesis, and Golgi/vesicular transport processes (Fig. 2D). In parallel, GSEA revealed significant enrichment changes in pathways related to proliferation, T-cell activation, and apoptosis (Fig. 2E). These findings support the notion that high ZUP1 expression coincides with coordinated alterations in epithelial structural/transport programs and immune-related transcriptional states, providing molecular clues for its involvement in resistance-associated phenotypes.

Before examining ZUP1 expression, we evaluated the cisplatin-resistant phenotypes of the cell models. The estimated cisplatin IC₅₀ increased from approximately 1.9 µM in parental A549 cells to 16.1 µM in A549/R cells and from approximately 4.2 µM in parental SK-MES-1 cells to 21.1 µM in SK-MES-1/R cells. The corresponding resistance indices were approximately 8.5 and 5.0, respectively (Supplementary Fig. S1A–B). These results supported reduced cisplatin sensitivity in both resistant derivatives.To validate these transcriptomic signals in experimental models, we compared ZUP1 expression between cisplatin-resistant sublines and their parental counterparts. qRT–PCR showed significantly higher ZUP1 mRNA expression in A549/R and SK-MES-1/R cells than in their corresponding parental A549 and SK-MES-1 cells, respectively (Fig. 2F). Consistent with the mRNA results, Western blot analysis demonstrated increased ZUP1 protein expression in both resistant derivatives. Densitometric analysis showed that ZUP1 protein expression was approximately 1.9-fold higher in A549/R cells and 2.3-fold higher in SK-MES-1/R cells than in their respective parental cells (Supplementary Fig. S1C–D). Taken together, the integrative screening, the association between high ZUP1 expression and poorer overall survival in the expanded public cohort, and the increased ZUP1 expression observed in cisplatin-resistant cell models supported the selection of ZUP1 as a candidate DUB for subsequent functional evaluation.

### ZUP1 knockdown suppresses growth of cisplatin-resistant NSCLC cells and promotes apoptosis

**Fig. 3:**
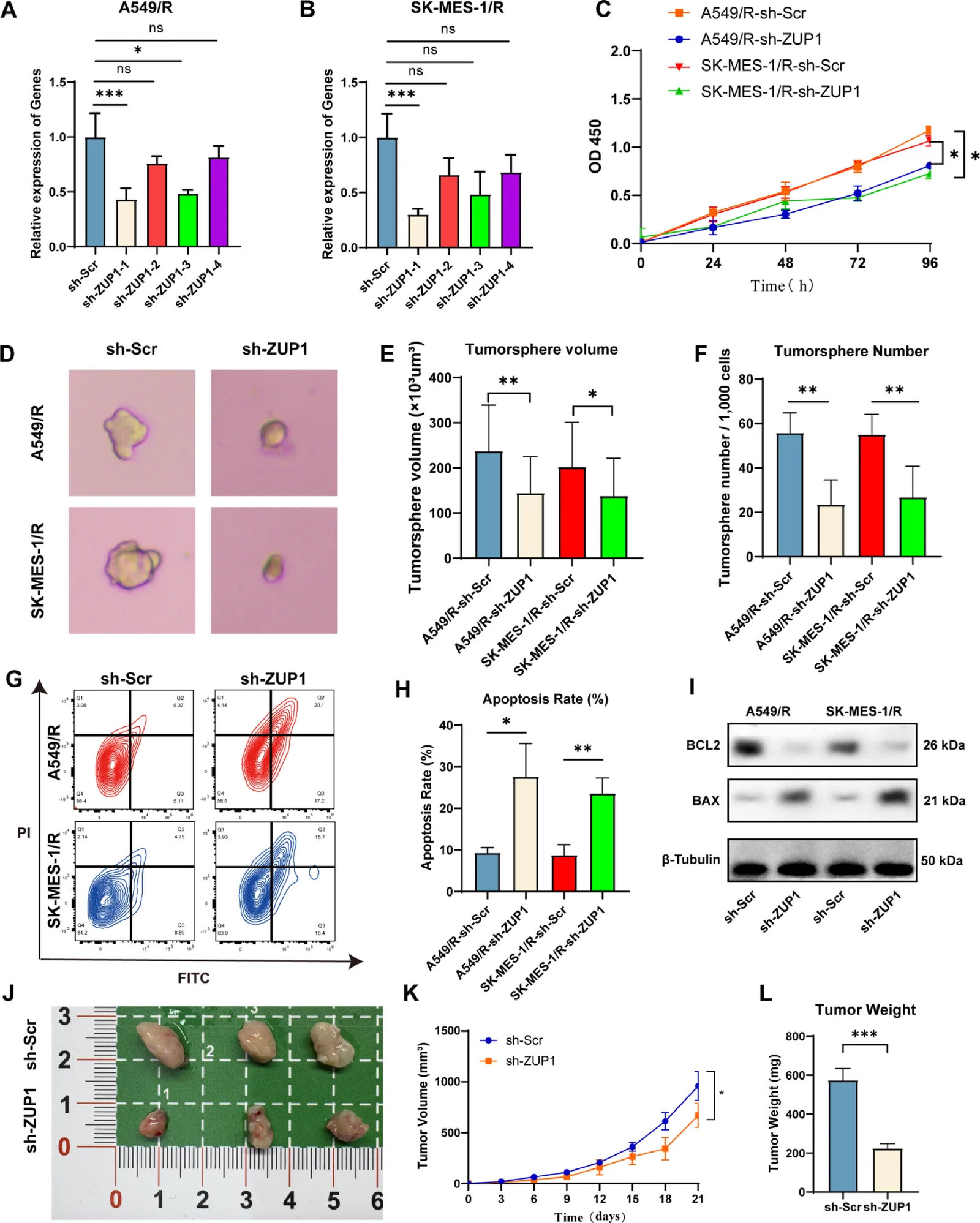
ZUP1 knockdown attenuates the growth of cisplatin-resistant NSCLC cells and increases apoptosis in vitro and in vivo. (**A, B**) Quantitative real-time PCR evaluation of three independent ZUP1-targeting shRNAs in A549/R (**A**) and SK-MES-1/R (**B**) cells. ZUP1 expression was normalized to GAPDH and expressed relative to the non-targeting shRNA control. shZUP1-1, which produced efficient and reproducible suppression in both cell lines, was selected for subsequent experiments. (**C**) CCK-8 growth curves of A549/R-sh-Scr, A549/R-sh-ZUP1, SK-MES-1/R-sh-Scr, and SK-MES-1/R-sh-ZUP1 cells measured at 0, 24, 48, 72, and 96 h after seeding. Cell growth was evaluated by measuring absorbance at 450 nm. (**D**) Representative images of tumorspheres formed by A549/R and SK-MES-1/R cells expressing sh-Scr or sh-ZUP1. Cells were seeded at 1×103 cells per well in ultra-low-attachment plates and cultured in serum-free tumorsphere medium for approximately 10 days. (**E**) Quantification of tumorsphere volume after approximately 10 days of culture. The diameter (d) of each tumorsphere was measured from microscopic images using ImageJ, and volume was estimated assuming spherical geometry using the formula V = πd³/6. Values are expressed in µm³. (**F**) Quantification of tumorsphere number in A549/R and SK-MES-1/R cells expressing sh-Scr or sh-ZUP1. Results are expressed as the number of tumorspheres formed per 1 × 10³ seeded cells. (**G**) Representative Annexin V–FITC/propidium iodide flow-cytometry plots of A549/R and SK-MES-1/R cells expressing sh-Scr or sh-ZUP1. Annexin V⁺/PI⁻ and Annexin V⁺/PI⁺ cells were classified as early apoptotic and late apoptotic/necrotic populations, respectively. (**H**) Quantification of the total apoptotic population, calculated as the sum of Annexin V⁺/PI⁻ and Annexin V⁺/PI⁺ cells. (**I**) Representative Western blot analysis of BCL2 and BAX expression in A549/R and SK-MES-1/R cells expressing sh-Scr or sh-ZUP1. β-Tubulin was used as the loading control. Corresponding densitometric analyses and uncropped blot images are provided in the Supplementary Information. (**J**) Representative images of excised subcutaneous xenograft tumors generated from A549/R cells stably expressing sh-Scr or sh-ZUP1. (**K**) Growth curves of A549/R-sh-Scr and A549/R-sh-ZUP1 xenografts. Female BALB/c nude mice aged 4–5 weeks were subcutaneously inoculated with 5×106 cells per mouse in 100 µL PBS/Matrigel mixture. Tumor volume was monitored for 21 days and calculated as (length×width2)/2. n = 3 mice per group. (**L**) Endpoint tumor weights of A549/R-sh-Scr and A549/R-sh-ZUP1 xenografts. n = 3 mice per group.Data are presented as mean ± SD from three independent biological experiments unless otherwise indicated. Panels (**A**) and (**B**) were analyzed using one-way ANOVA with an appropriate multiple-comparisons test. Time-course data in (**C**) and (**K**) were analyzed using two-way ANOVA. Comparisons in (**E**), (**F**), (**H**), and (**L**) were analyzed using two-tailed unpaired Student’s t-tests. ns, not significant; ∗P < 0.05;∗∗P < 0.01;∗∗∗P < 0.001.

To determine the functional contribution of ZUP1 to the cisplatin-resistant phenotype, we established multiple shRNA-mediated knockdown systems in two representative cisplatin-resistant NSCLC cell lines, A549/R and SK-MES-1/R. qRT–PCR screening showed that several ZUP1-targeting shRNAs effectively reduced ZUP1 mRNA expression, and shZUP1-1 was selected because it produced efficient and reproducible knockdown in both cell lines (Fig. 3A, B). Western blotting and densitometric analysis further confirmed that the selected shZUP1 construct significantly reduced ZUP1 protein expression in A549/R and SK-MES-1/R cells compared with their corresponding sh-Scr groups (Supplementary Fig. S2A, B). These results validated the stable ZUP1-knockdown models used in the subsequent functional assays. In proliferation assays, longitudinal CCK-8 measurements showed that ZUP1 knockdown markedly slowed the growth of both resistant cell lines, with the most pronounced differences observed at 72–96 h (Fig. 3C).After approximately 10 days of tumorsphere culture, ZUP1-knockdown A549/R and SK-MES-1/R cells formed smaller tumorspheres than their corresponding sh-Scr controls, and quantitative analysis showed a reduction in the estimated tumorsphere volume following ZUP1 knockdown (Fig. 3D–E).To determine whether these effects were restricted to cisplatin-resistant cells, we performed corresponding experiments in parental A549 and SK-MES-1 cells. ZUP1 knockdown significantly reduced the estimated tumorsphere volume and tumorsphere number in both parental cell lines (Supplementary Fig. S3A–C). ALDH1A1 expression was significantly reduced in both A549 and SK-MES-1 cells. PROM1 expression was significantly reduced in A549 cells but not in SK-MES-1 cells, whereas CD44 expression was significantly reduced in SK-MES-1 cells but not in A549 cells (Supplementary Fig. S3D). These findings indicate that the effects of ZUP1 knockdown on growth and selected stemness-associated phenotypes are not restricted to cisplatin-resistant cells, although the marker-expression responses show cell-line-dependent heterogeneity. Consistent with this phenotype, qRT–PCR analysis showed that the mRNA expression of the stemness-associated markers ALDH1A1, PROM1/CD133, and CD44 was significantly reduced in both resistant cell lines following ZUP1 knockdown (Supplementary Fig. S3E). Together, these results suggest that ZUP1 knockdown is associated with attenuated stemness-related phenotypes in cisplatin-resistant NSCLC cells.

Quantification of tumorsphere number further showed that ZUP1 knockdown significantly decreased the number of tumorspheres formed per 1 × 10³ seeded cells, indicating reduced tumorsphere-forming capacity (Fig. 3F). Annexin V/PI flow cytometry showed that ZUP1 knockdown increased the proportions of early and late apoptotic cells in both resistant cell lines (Fig. 3G–H). Consistent with this finding, Western blot analysis showed reduced BCL2 expression and increased BAX expression following ZUP1 knockdown (Fig. 3I). These apoptosis-related protein changes were consistent with the increased apoptotic phenotype detected by flow cytometry.

The growth-inhibitory effect of ZUP1 knockdown was further evaluated in vivo. In the subcutaneous xenograft model, tumors derived from A549/R-shZUP1 cells grew more slowly and exhibited lower endpoint tumor weights than tumors derived from control cells (Fig. 3J–L). No significant difference in body weight was observed between the two groups during the experimental period, and Western blot analysis of the excised tumor tissues confirmed reduced ZUP1 expression in the shZUP1 group (Supplementary Fig. S4A–C).Collectively, these findings suggest that ZUP1 contributes to the growth, tumorsphere-forming capacity, and survival of cisplatin-resistant NSCLC cells. These results support further investigation of ZUP1 as a candidate therapeutic target in cisplatin-resistant NSCLC.

### ZUP1 expression is associated with epithelial-to-treg signaling and treg-related phenotypes in resistant NSCLC

**Fig. 4:**
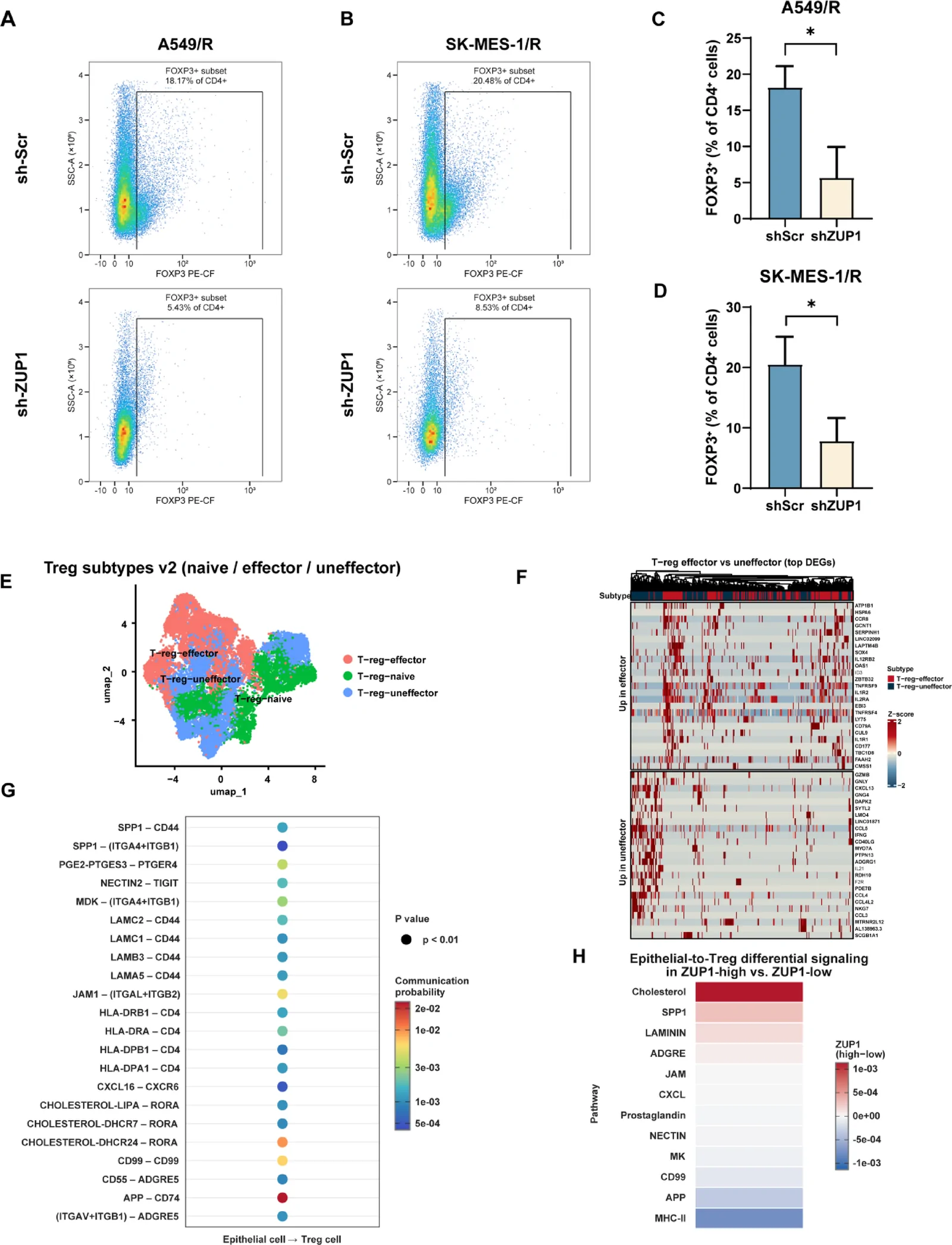
Tumor-cell ZUP1 is associated with an increased CD4⁺FOXP3⁺ Treg phenotype and altered epithelial-to-Treg communication in cisplatin-resistant NSCLC. (**A, B**) Representative flow-cytometry plots showing FOXP3 expression within the gated CD4⁺ population recovered following co-culture with A549/R (**A**) or SK-MES-1/R (**B**) cells stably expressing sh-Scr or sh-ZUP1. Lymphocytes and singlets were sequentially gated before establishing the CD4⁺ parent population. Numbers indicate the percentage of FOXP3⁺ cells among total gated CD4⁺ cells. (**C, D**) Quantification of FOXP3⁺ cells within the total gated CD4⁺ population following co-culture with A549/R (**C**) or SK-MES-1/R (**D**) cells expressing sh-Scr or sh-ZUP1. Data are presented as mean ± SD from three independent biological experiments. Comparisons were performed using two-tailed unpaired Student’s t-tests. ∗P < 0.05. (**E**) UMAP visualization of Treg cells extracted and re-clustered from the integrated NSCLC single-cell datasets GSE207422 and GSE243013. Cells were annotated as naïve, effector, or uneffector Treg subsets according to their transcriptional profiles and the marker criteria described in the Methods. (**F**) Heatmap showing representative differentially expressed genes between effector and uneffector Treg subsets. Columns represent individual cells and rows represent selected genes. Expression values were scaled by gene and are displayed as Z scores; red and blue indicate relatively high and low expression, respectively. (**G**) CellChat-inferred ligand–receptor interactions from epithelial cells to Tregs in the integrated single-cell dataset. Each row represents a predicted ligand–receptor pair. Dot color indicates the inferred communication probability, with warmer colors representing higher probabilities. Only statistically supported interactions with P < 0.01 are shown. (**H**) Differential pathway-level signaling from ZUP1-high versus ZUP1-low epithelial cells to Tregs. Values were calculated as the inferred communication strength from ZUP1-high epithelial cells minus that from ZUP1-low epithelial cells. Red indicates stronger signaling from ZUP1-high epithelial cells, whereas blue indicates stronger signaling from ZUP1-low epithelial cells. Data in (**C**) and (**D**) are presented as mean ± SD from three independent co-culture experiments. Statistical significance was assessed using a two-tailed unpaired Student’s t-test. ∗P < 0.05;∗∗P < 0.01.

Given the altered intercellular communication patterns observed in the Resistant dataset, we next investigated whether tumor-cell ZUP1 was associated with Treg-related phenotypes and epithelial–immune communication.In both the A549/R and SK-MES-1/R co-culture settings, sequential flow-cytometry analysis using total CD4⁺ cells as the parent population showed that the proportion of FOXP3⁺ cells among CD4⁺ cells was significantly lower following co-culture with ZUP1-knockdown A549/R and SK-MES-1/R cells than following co-culture with the corresponding sh-Scr control cells (Fig. 4A–D). Representative analyses showed reductions from 18.17% to 5.43% in the A549/R system and from 20.48% to 8.53% in the SK-MES-1/R system. These findings indicate that the reduced FOXP3⁺ fraction was retained after normalization to the total CD4⁺ population.

To further define the transcriptional features underlying Treg state changes at single-cell resolution, we subdivided Tregs into naïve, effector, and uneffector subsets and compared their expression profiles. Distinct differential gene signatures were observed between effector and uneffector Tregs, with effector Tregs showing stronger enrichment of activation/suppressive programs, which were more pronounced in the resistance-associated context (Fig. 4E–F). We then integrated CellChat analyses to quantify ligand–receptor communication from tumor epithelial cells to Tregs and to trace the interaction basis for these observations. Compared with ZUP1-low epithelial cells, ZUP1-high epithelial cells exhibited globally increased outgoing signaling toward Tregs and showed enrichment in multiple pathways linked to immunosuppression, adhesion/residency, and metabolism (Fig. 4G–H). Representative pathways included SPP1- and laminin-associated axes, JAM/NECTIN-mediated interactions, and MHC-II-related signaling. These predicted interactions suggest that ZUP1-high epithelial cells exhibit an altered signaling profile toward Tregs; however, the functional contributions of the individual ligand–receptor pathways remain to be experimentally determined.

To identify candidate tumor-derived factors potentially associated with the Treg-related phenotype, we examined SPP1, CXCL16, and PTGS2, which were implicated in the epithelial-to-Treg communication analysis. ZUP1 knockdown significantly reduced SPP1 and CXCL16 mRNA expression in both A549/R and SK-MES-1/R cells. PTGS2 expression was reduced in A549/R cells, whereas the change in SK-MES-1/R cells did not reach statistical significance (Supplementary Fig. S5A–C).

ELISA analysis further showed that the amount of SPP1 secreted into the conditioned medium, normalized to viable cell number, was significantly reduced following ZUP1 knockdown in both resistant cell lines (Supplementary Fig. S5D). Together with the predicted SPP1–CD44 and SPP1–integrin-associated interactions, these findings suggest that reduced tumor-cell SPP1 secretion may represent one candidate link between ZUP1 knockdown and the altered CD4⁺FOXP3⁺ cell fraction observed in the co-culture system. However, direct causality remains to be established using SPP1 rescue, neutralization, or receptor-blocking experiments.

Together, the co-culture and cell-communication analyses suggest an association between tumor-cell ZUP1 expression and Treg-related phenotypes in resistant NSCLC. Further mechanistic experiments will be required to determine whether specific epithelial-to-Treg signaling pathways directly mediate this relationship.

### Peimisine binds ZUP1 and inhibits the growth of cisplatin-resistant NSCLC cells

**Fig. 5:**
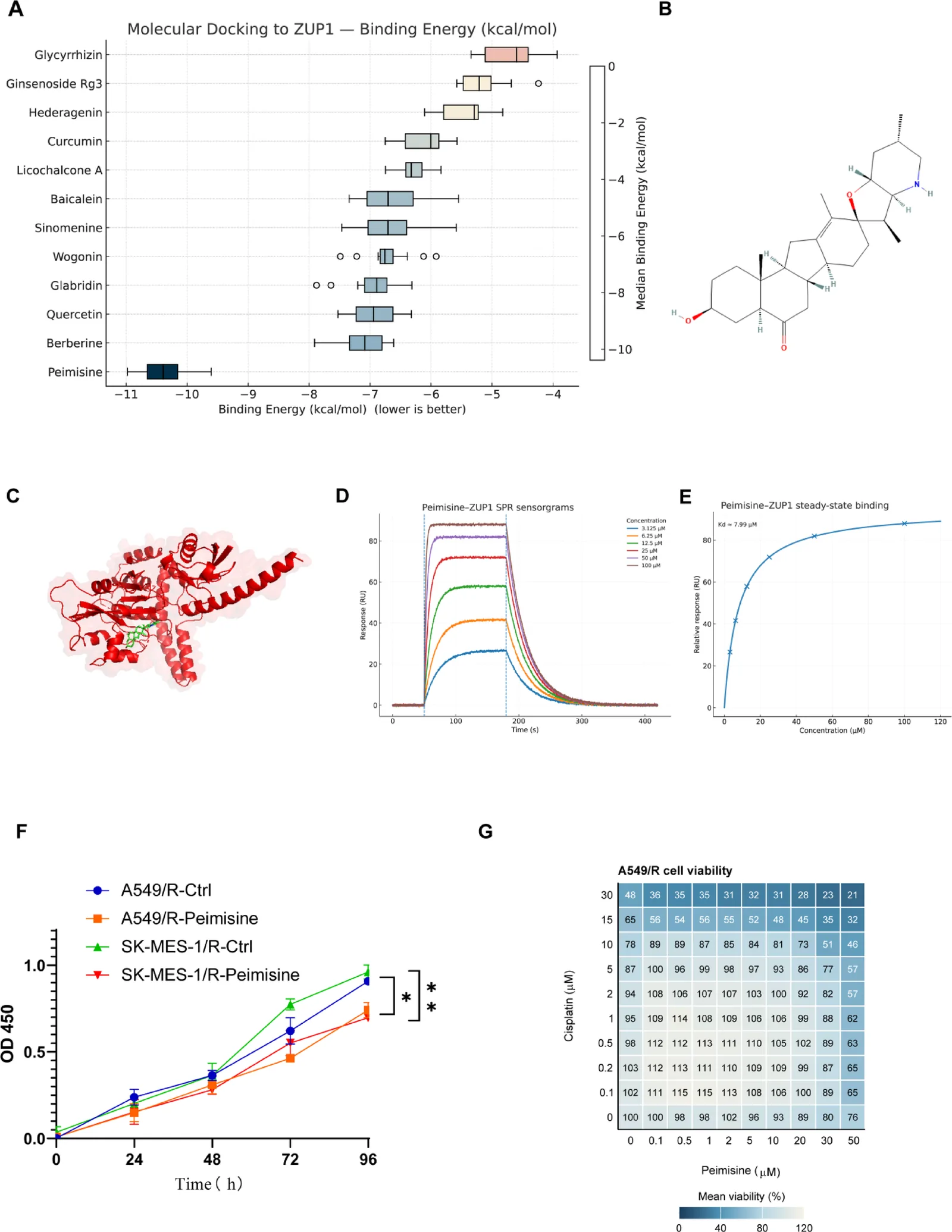
Peimisine directly binds ZUP1 and inhibits the growth of cisplatin-resistant NSCLC cells, with concentration-dependent interactions with cisplatin. (**A**) Molecular-docking analysis comparing the predicted binding energies of selected natural compounds within the predicted ZUP1 binding pocket. Box plots show the distributions of docking scores obtained for the evaluated poses. More negative values indicate more favorable predicted binding, and peimisine showed the lowest predicted binding energy among the tested compounds. (**B**) Two-dimensional chemical structure of peimisine. (**C**) Representative predicted binding pose of peimisine within the ZUP1 binding pocket. ZUP1 is shown in ribbon and surface representations, and peimisine is shown in stick representation. (**D**) Surface plasmon resonance sensorgrams showing the concentration-dependent association and dissociation of peimisine with immobilized recombinant human ZUP1. Peimisine was injected at 3.125, 6.25, 12.5, 25, 50, and 100 µM. Responses from the reference channel were subtracted before analysis. (**E**) Steady-state binding analysis of the peimisine–ZUP1 interaction. The fitted binding curve yielded an estimated equilibrium dissociation constant of K d = 7.99 µM. (**F**) CCK-8 growth curves of A549/R and SK-MES-1/R cells treated with vehicle control or 50 µM peimisine and analyzed at 0, 24, 48, 72, and 96 h. Cell growth was evaluated by measuring absorbance at 450 nm. (**G**) Heatmap showing normalized cell viability of A549/R cells following treatment with the indicated concentration combinations of peimisine and cisplatin. Each tile represents the mean viability from three independent biological experiments for one experimentally tested dose combination. Data in (**F**) are presented as mean ± SD from three independent biological experiments and were analyzed using two-way ANOVA. SPR experiments were independently repeated at least three times. ∗P < 0.05;∗∗P < 0.01.

To explore a translational intervention strategy, we performed natural-product screening with ZUP1 as the target. Molecular docking was first used to estimate the binding energies of candidate natural compounds to ZUP1. Peimisine ranked among the top candidates and showed the most favorable predicted binding energy (approximately − 11 kcal/mol) (Fig. 5A), and its chemical structure is shown in Fig. 5B. Docking poses indicated that peimisine could stably occupy the ZUP1 binding pocket and form multiple interactions within the cavity (Fig. 5C), providing a structural rationale for its potential as a candidate ZUP1-binding natural compound.

We next validated direct binding at the biochemical level using surface plasmon resonance (SPR). Dose-series SPR sensorgrams exhibited reversible association and dissociation kinetics (Fig. 5D), and steady-state fitting yielded micromolar affinity (K_d ≈ 7.99 µM) (Fig. 5E), consistent with the docking-based prioritization and supporting direct interaction between peimisine and ZUP1.

Functionally, peimisine inhibited the growth of cisplatin-resistant A549/R and SK-MES-1/R cells over time (Fig. 5F). Comparison with the corresponding parental cell lines showed a modestly greater reduction in viability in the resistant derivatives following peimisine treatment (Supplementary Fig. S6 A-B). However, because the target dependence of this differential response was not examined, this observation was not interpreted as ZUP1-dependent selectivity.

We next directly examined the experimentally measured viability of A549/R cells across a two-dimensional concentration matrix of peimisine and cisplatin. Cell viability progressively decreased under selected combination conditions (Fig. 5G). To account for the baseline effect of peimisine, cisplatin responses at fixed peimisine concentrations were normalized to the corresponding peimisine-only controls within each replicate. The estimated cisplatin IC₅₀ decreased from 28.3 µM in the absence of peimisine to 14.9 and 12.2 µM in the presence of 30 and 50 µM peimisine, respectively (Supplementary Fig. S6C). These findings indicate that peimisine enhanced the in vitro cisplatin response in the tested A549/R model. Further studies are required to establish whether this effect is mediated through ZUP1 and whether it extends to additional resistant models and in vivo treatment settings.

## Discussion

Platinum-based chemotherapy remains an important component of systemic treatment for non–small cell lung cancer (NSCLC), but the development of acquired resistance substantially limits its long-term efficacy. Cisplatin resistance is a multifactorial process involving tumor-cell-intrinsic adaptations, including enhanced damage tolerance, impaired apoptotic responses, and the maintenance of stemness-associated phenotypes, as well as changes in the tumor microenvironment that may support the persistence of resistant cells^19,20^. In the present study, we integrated outcome-associated single-cell analyses, public-cohort evaluation, and experimental validation to identify ZUP1 as a candidate factor associated with cisplatin-resistant NSCLC. ZUP1 expression was increased at both the mRNA and protein levels in A549/R and SK-MES-1/R cells, and high ZUP1 expression was associated with poorer overall survival in the expanded public NSCLC cohort. Functionally, ZUP1 knockdown reduced cell growth, tumorsphere number and volume, and xenograft growth, while increasing apoptosis. Together, these findings support the involvement of ZUP1 in maintaining growth- and survival-related properties in cisplatin-resistant NSCLC cells, although they do not establish ZUP1 as the sole determinant of resistance.

The increased apoptosis following ZUP1 knockdown was accompanied by reduced BCL2 and increased BAX expression, indicating that the observed growth inhibition was associated with a shift toward a pro-apoptotic state. ZUP1 knockdown also reduced tumorsphere growth and decreased the expression of the stemness-associated markers ALDH1A1, PROM1/CD133, and CD44 in the resistant cell lines. Apoptosis avoidance and stemness-associated plasticity are both recognized contributors to the persistence and repopulation of tumor cells during chemotherapy^19,20^. However, the additional experiments using parental A549 and SK-MES-1 cells showed that ZUP1 knockdown also reduced tumorsphere formation and growth, as well as selected stemness-associated markers in non-resistant cells. The effects of ZUP1 are therefore not strictly restricted to the cisplatin-resistant state. Nevertheless, the consistently higher ZUP1 expression in both resistant derivatives, together with the marked functional effects observed in A549/R and SK-MES-1/R cells, suggests that ZUP1 may contribute to the maintenance of the resistant phenotype.

Our single-cell analyses further revealed differences in cellular composition and predicted intercellular communication between post-treatment resistant and remission NSCLC. We restricted the immune-regulatory interpretation to the Treg-focused analyses. Tregs can exhibit enhanced suppressive activity in NSCLC, and their accumulation in specific tumor compartments has been associated with impaired antitumor immunity and unfavorable clinical outcomes^21,22^. In our study, ZUP1-high epithelial cells showed stronger predicted outgoing communication toward Tregs, whereas ZUP1 knockdown in resistant tumor cells was associated with a reduced CD4⁺FOXP3⁺ cell fraction in the co-culture system. These complementary observations suggest an association between tumor-cell ZUP1 expression and Treg-related phenotypes, but do not by themselves demonstrate that ZUP1 directly induces Treg differentiation or activation.

Among the candidate tumor-derived mediators identified by the epithelial-to-Treg communication analysis, ZUP1 knockdown consistently reduced SPP1 and CXCL16 mRNA expression in both resistant cell lines, whereas the effect on PTGS2 was cell-line dependent. Secreted SPP1, normalized to viable tumor-cell number, was also reduced following ZUP1 knockdown in both A549/R and SK-MES-1/R cells. SPP1, also known as osteopontin, is an extracellular and immunomodulatory protein that can interact with CD44 and integrin receptors and has been implicated in the establishment of tumor-supportive immune environments^23^. More recent evidence also indicates that osteopontin contributes to the stability and suppressive function of FOXP3⁺ Tregs^24^. These observations provide biological context for the SPP1–CD44 and SPP1–integrin interactions predicted in our analysis. We therefore propose that reduced tumor-cell SPP1 secretion may represent one candidate link between ZUP1 knockdown and the altered CD4⁺FOXP3⁺ cell fraction observed in the co-culture system. This interpretation remains provisional, because SPP1 supplementation, neutralization, and receptor-blocking experiments were not performed. Similar functional validation will also be required for the other predicted ligand–receptor pathways, including CXCL16-, laminin-, JAM-, NECTIN-, and MHC-II-associated signaling.

ZUP1 belongs to a structurally distinct class of deubiquitinating enzymes and preferentially cleaves K63-linked polyubiquitin chains^25,26^. Previous studies have linked ZUP1 to replication-associated stress responses and genome stability, including regulation of the replication protein A complex and the resolution of K63-linked ubiquitin signals^25,26^. Because cisplatin-induced DNA adducts interfere with DNA replication and generate replication stress, these established biochemical properties provide a plausible context in which increased ZUP1 expression could support cellular tolerance to cisplatin-induced stress. In addition, ZUP1 has recently been implicated in MAVS-associated innate immune signaling, suggesting that its biological functions may extend beyond genome maintenance^27^. Other deubiquitinases have also been shown to regulate tumor immune signaling by controlling the stability of immune-related proteins, including PD-L1^28^. Nevertheless, our present experiments did not directly examine DNA-damage repair, replication-fork stability, innate immune signaling, or ZUP1 substrates. The proposed connection between the known biochemical functions of ZUP1 and the resistant and Treg-related phenotypes observed here should therefore be considered a working model rather than a demonstrated mechanism.

From a translational perspective, our natural-product screening identified peimisine as a compound capable of directly binding recombinant ZUP1. SPR analysis indicated a micromolar binding affinity, with an estimated equilibrium dissociation constant of approximately 7.99 µM. Peimisine inhibited the growth of both A549/R and SK-MES-1/R cells, and comparison with the corresponding parental cells showed a modestly greater reduction in viability in the resistant derivatives. However, because ZUP1 depletion or rescue was not used to assess peimisine sensitivity, this difference cannot currently be interpreted as ZUP1-dependent selectivity. In the A549/R combination model, the estimated cisplatin IC₅₀ decreased from 28.3 µM in the absence of peimisine to 14.9 and 12.2 µM at fixed peimisine concentrations of 30 and 50 µM, respectively. These results indicate that peimisine enhanced the in vitro cisplatin response under the tested conditions. However, because the combination analysis was restricted to one resistant cell model and the effects varied across the tested concentration range, the present data do not establish broad pharmacological synergy or demonstrate that peimisine reverses cisplatin resistance through ZUP1. Peimisine is therefore more appropriately regarded as a preliminary ZUP1-binding natural-product lead that warrants further target-dependence, structure–activity, pharmacological, and combination-treatment studies.

Several limitations of this study should be acknowledged. First, although independent shRNA experiments consistently linked ZUP1 knockdown to reduced tumor-cell growth and increased apoptosis, ZUP1 re-expression and catalytic-mutant rescue experiments were not performed. It therefore remains unknown whether the observed phenotypes specifically depend on the deubiquitinase activity of ZUP1. Second, the relevant ZUP1 substrates and downstream signaling pathways were not identified. Third, the relationship between tumor-cell ZUP1 and Treg-related phenotypes was inferred from computational communication analysis and an in vitro co-culture system. Individual ligand–receptor pathways were not validated by blocking or rescue experiments, and the immunodeficient xenograft model could not assess the contribution of an intact immune microenvironment. Fourth, the single-cell comparison incorporated public datasets derived from different patient cohorts, and residual cohort- or treatment-related heterogeneity may have influenced the observed differences. Finally, although peimisine directly bound ZUP1 in the SPR assay, its cellular target dependence, pharmacokinetic properties, in vivo efficacy, toxicity, and therapeutic interaction with cisplatin remain to be determined.

## Conclusion

Starting from outcome-linked single-cell datasets, this study revealed differences in cellular composition and predicted immune-related intercellular communication between resistant and remission NSCLC, together with a shift of epithelial cells toward a communication-hub state.Functional experiments demonstrate that inhibiting ZUP1 suppresses proliferation, tumorsphere formation of cisplatin-resistant cells, promotes apoptosis, and reduces tumor growth in vivo. In parallel, ZUP1 knockdown was associated with altered epithelial-to-Treg signaling and a lower FOXP3⁺ fraction within the gated CD4⁺ population in co-culture; whether this relationship is causal remains to be established. Translationally, the natural product peimisine directly binds ZUP1 and inhibits resistant-cell growth. In A549/R cells, fixed peimisine concentrations were associated with lower estimated cisplatin IC₅₀ values, supporting further investigation of peimisine as a preliminary ZUP 1-binding natural-product lead. 

## Supplementary Information

Below is the link to the electronic supplementary material.

Supplementary Material 1

Supplementary Material 2

## Data Availability

The public single-cell RNA-sequencing datasets analyzed in this study are available in the NCBI Gene Expression Omnibus under accession numbers GSE207422 and GSE243013. The gene-expression and clinical data used for the pooled survival analysis were obtained from cBioPortal for Cancer Genomics. All data generated or analyzed during this study are included in this article and its Supplementary Information files. Additional data supporting the findings of this study are available from the corresponding author, Nan Yan, upon reasonable request.

## Code availability

No custom software or algorithms were developed in this study. The analyses were performed using publicly available software and R packages as described in the Materials and Methods.
