# Supplementary material for: ZUP1 contributes to cisplatin resistance and treg-related signaling in non–small cell lung cancer
Source: Sci Rep. 2026 Sep 10;16:28346. doi: 10.1038/s41598-026-70626-y (PMC13562685; doi:10.1038/s41598-026-70626-y)
Supplement: Supplementary file 1 [file 41598_2026_70626_MOESM1_ESM.docx]

**Supplementary figure legends**

**Supplementary Figure S1 | Validation of cisplatin resistance and increased ZUP1 protein expression in cisplatin-resistant NSCLC cells.**

(A, B) Cisplatin dose–response curves for parental A549 and A549/R cells (A) and parental SK-MES-1 and SK-MES-1/R cells (B). Cells were treated with the indicated concentrations of cisplatin, and cell viability was measured using the CCK-8 assay. Viability was normalized to the corresponding untreated group.

(C) Representative Western blot analysis of ZUP1 protein expression in parental A549 and SK-MES-1 cells and their corresponding cisplatin-resistant derivatives, A549/R and SK-MES-1/R. GAPDH was used as the loading control.

(D) Densitometric quantification of ZUP1 protein expression in A549 versus A549/R and SK-MES-1 versus SK-MES-1/R cells. ZUP1 signals were normalized to GAPDH and expressed relative to the corresponding parental cell line.

Data are presented as mean ± SD from three independent experiments. Cisplatin dose–response curves were analyzed using two-way ANOVA, and Western blot quantifications were analyzed using two-tailed unpaired Student’s t-tests. ∗P<0.05;∗∗P<0.01.

**Supplementary Figure S2 | Protein-level validation of ZUP1 knockdown and apoptosis-related protein changes in cisplatin-resistant NSCLC cells.**

(A) Representative Western blot analysis of ZUP1 expression in A549/R and SK-MES-1/R cells stably expressing sh-Scr or sh-ZUP1. GAPDH was used as the loading control.

(B) Densitometric quantification of ZUP1 protein expression. ZUP1 signals were normalized to GAPDH and expressed relative to the corresponding sh-Scr group.

(C) Densitometric quantification of BAX protein expression. BAX signals were normalized to β-Tubulin and expressed relative to the corresponding sh-Scr group.

(D) Densitometric quantification of BCL2 protein expression. BCL2 signals were normalized to β-Tubulin and expressed relative to the corresponding sh-Scr group.

Data are presented as mean ± SD from three independent biological experiments. Statistical significance was assessed using two-tailed unpaired Student’s t-tests. *P < 0.05; **P < 0.01; ***P < 0.001.

**Supplementary Figure S3 | Effects of ZUP1 knockdown on tumorsphere growth and formation and stemness-associated gene expression in parental and cisplatin-resistant NSCLC cells.**

(A) Representative images of tumorspheres generated by parental A549 and SK-MES-1 cells stably expressing sh-Scr or sh-ZUP1. Cells were seeded at 1×10^3^ cells per well in ultra-low-attachment plates and cultured in serum-free tumorsphere medium for approximately 10 days.

(B)Quantification of tumorsphere volume in parental A549 and SK-MES-1 cells expressing sh-Scr or sh-ZUP1. The diameter (d) of each tumorsphere was measured from microscopic images using ImageJ, and volume was estimated assuming spherical geometry using the formula V = πd³/6. Values are expressed in μm³.

(C) Quantification of tumorsphere number in parental A549 and SK-MES-1 cells expressing sh-Scr or sh-ZUP1. Results are expressed as the number of tumorspheres formed per 1 × 10³ seeded cells.

(D) Quantitative real-time PCR analysis of the stemness-associated genes ALDH1A1, PROM1/CD133, and CD44 in parental A549 and SK-MES-1 cells expressing sh-Scr or sh-ZUP1.

(E) Quantitative real-time PCR analysis of ALDH1A1, PROM1/CD133, and CD44 in cisplatin-resistant A549/R and SK-MES-1/R cells expressing sh-Scr or sh-ZUP1.Gene expression was normalized to GAPDH using the 2−ΔΔCt method and expressed relative to the corresponding sh-Scr group. Data are presented as mean ± SD from three independent biological experiments. Statistical significance was assessed using two-tailed unpaired Student’s t-tests for comparisons between sh-Scr and sh-ZUP1 within each cell line. ns, not significant; ∗P<0.05;∗∗P<0.01;∗∗∗P<0.001.

**Supplementary Figure S4 | Body-weight monitoring and validation of sustained ZUP1 knockdown in A549/R xenograft tumors.**

(A) Body-weight curves of BALB/c nude mice bearing subcutaneous A549/R-sh-Scr or A549/R-sh-ZUP1 xenografts during the 21-day experimental period. n=3 mice per group.

(B) Representative Western blot analysis of ZUP1 expression in xenograft tumor tissues collected at the experimental endpoint. GAPDH was used as the loading control.

(C) Densitometric quantification of ZUP1 protein expression in A549/R-sh-Scr and A549/R-sh-ZUP1 xenograft tumors. ZUP1 signals were normalized to GAPDH and expressed relative to the sh-Scr group.

Data are presented as mean ± SD. Body-weight curves were analyzed using two-way ANOVA, and endpoint ZUP1 expression was analyzed using a two-tailed unpaired Student’s t-test. ns, not significant; ∗P<0.05.

**Supplementary Figure S5 | ZUP1 knockdown reduces the expression and secretion of Treg-associated signaling factors in cisplatin-resistant NSCLC cells.**

(A–C) Quantitative real-time PCR analysis of SPP1 (A), CXCL16 (B), and PTGS2 (C) expression in A549/R and SK-MES-1/R cells stably expressing sh-Scr or sh-ZUP1. Gene expression was normalized to GAPDH using the 2

−ΔΔCt method and expressed relative to the corresponding sh-Scr group.

(D) Enzyme-linked immunosorbent assay measurement of secreted SPP1 in conditioned medium collected from A549/R and SK-MES-1/R cells expressing sh-Scr or sh-ZUP1. SPP1 concentrations were normalized to the number of viable tumor cells and are expressed as pg per 10^6^ viable cells.

Data are presented as mean ± SD from three independent biological experiments. Statistical significance was assessed using two-tailed unpaired Student’s t-tests for comparisons between sh-Scr and sh-ZUP1 within each cell line. ns, not significant; ∗P<0.05;∗∗P<0.01;∗∗∗P<0.001.

**Supplementary Figure S6 | Comparison of peimisine sensitivity between parental and cisplatin-resistant NSCLC cells.**

(A) Peimisine dose–response curves for parental A549 and cisplatin-resistant A549/R cells.

(B) Peimisine dose–response curves for parental SK-MES-1 and cisplatin-resistant SK-MES-1/R cells.

Cells were exposed to the indicated concentrations of peimisine, and cell viability was measured using the CCK-8 assay. Viability was normalized to the corresponding vehicle-treated group. Data are presented as mean ± SD from three independent experiments. Statistical significance was assessed using two-way ANOVA. ∗∗P<0.01.

(C) Cisplatin dose–response curves in A549/R cells in the presence of 0, 30, or 50 μM peimisine. Within each independent experiment, viability under combination treatment was normalized to the corresponding peimisine-only condition. Curves were fitted using a constrained four-parameter log-logistic model. The estimated cisplatin IC₅₀ values were 28.3, 14.9, and 12.2 μM, respectively; 95% confidence intervals are shown.

**Supplementary table legends**

**Supplementary Table S1 | Primer and shRNA sequences used in this study.**
All oligonucleotide sequences are presented in the 5′–3′ direction. qPCR, quantitative real-time polymerase chain reaction; shRNA, short hairpin RNA.

**Supplementary Table S2 | Differentially expressed genes between Resistant and Remission epithelial cells.**
Differential expression was assessed using the Wilcoxon rank-sum test. Positive average log₂ fold-change (log₂FC) values indicate higher expression in Resistant epithelial cells, whereas negative values indicate higher expression in Remission epithelial cells. Significant differentially expressed genes were defined by |average log₂FC| ≥ 0.25 and a Benjamini–Hochberg false discovery rate (BH FDR) < 0.05.

**Supplementary Table S3 | GO Biological Process enrichment of genes upregulated in Resistant epithelial cells.**
GO Biological Process over-representation analysis was performed using genes significantly upregulated in Resistant epithelial cells, defined by an average log₂FC ≥ 0.25 and BH FDR < 0.05. All genes tested in the differential-expression analysis were used as the background universe. Gene count indicates the number of upregulated genes assigned to each GO term. Only GO terms with BH FDR < 0.05 are presented. GO, Gene Ontology; FDR, false discovery rate.

**Supplementary Table S4 | GO Biological Process enrichment of genes upregulated in Remission epithelial cells.**
GO Biological Process over-representation analysis was performed using genes significantly upregulated in Remission epithelial cells, defined by an average log₂FC ≤ −0.25 and BH FDR < 0.05. All genes tested in the differential-expression analysis were used as the background universe. Gene count indicates the number of upregulated genes assigned to each GO term. Only GO terms with BH FDR < 0.05 are presented. GO, Gene Ontology; FDR, false discovery rate.
